# Prevalence of Non-Alcoholic Fatty Pancreas Disease (NAFPD) and its risk factors among adult medical check-up patients in a private hospital: a large cross sectional study

**DOI:** 10.1186/s12876-015-0404-1

**Published:** 2015-12-12

**Authors:** Cosmas Rinaldi A. Lesmana, Levina S. Pakasi, Sri Inggriani, Maria L. Aidawati, Laurentius A. Lesmana

**Affiliations:** 1 Digestive Disease & GI Oncology Center, Medistra Hospital, Jakarta, Indonesia; 2 Department of Internal Medicine, Hepatobiliary Division, Cipto Mangunkusumo Hospital, University of Indonesia, Jakarta, Indonesia; 3 Department of Radiology, Medistra Hospital, Jakarta, Indonesia

**Keywords:** Fatty pancreas, Metabolic syndrome, Non-alcoholic fatty pancreas disease, Risk factors

## Abstract

**Background:**

The clinical significance of non-alcoholic fatty pancreatic disease (NAFPD) or fatty pancreas is largely unknown. It is often an incidental finding on abdominal ultrasound, which is not explored further, especially its association with metabolic condition and the risk of pancreatic malignancy. The aim of this study is to evaluate the presence of NAFPD and its associated risk factors among adult medical check-up patients.

**Method:**

A large cross-sectional study was done among adult medical check-up patients underwent abdominal ultrasound between January and December 2013 in Medistra Hospital, Jakarta. Data was obtained from the patients’ medical record and include demographic data, blood pressures, fasting blood glucose level, and lipid profile. The presence of fatty pancreas was diagnosed by ultrasound. Bivariate and multivariate analyses were done to find associated risk factors for NAFPD. Statistical analysis was done using SPSS version 17.

**Results:**

A total of 1054 cases were included in this study; pancreas cannot be visualized in 153 cases and were excluded from the analysis. Fatty pancreas was present in 315 (35.0 %) patients. Bivariate analyses found associations among fatty pancreas and several risk factors such as gender, age, systolic and diastolic blood pressures, body mass index (BMI), fasting plasma glucose (FPG), triglycerides (TG) and cholesterol levels.

**Conclusion:**

Fatty pancreas is a common finding during medical check-up with a prevalence of 35 %. Fatty pancreas has significant association with metabolic factors and it might have an important role in risk of malignancy.

## Background

High energy intake in human may lead to excessive fat which could be accumulated in visceral organs that are unusual for adipose tissue storage, the so-called ectopic fat [1]. Fatty pancreas or nonalcoholic fatty pancreatic disease (NAFPD) is an excessive fat infiltration of the pancreas due to obesity in the absence of significant alcohol intake [2]. Fatty pancreas is a common ultrasound finding which has increased echogenicity when compared to the normal pancreas [3].

On the contrary to the nonalcoholic fatty liver disease (NAFLD), the potential systemic and local consequences of excessive fat accumulation in the pancreas have not been well established. Fatty infiltration in the pancreas has been showed to correlate with the metabolic risk factors and may represent a meaningful manifestation of metabolic syndrome., [4, 5] Epidemiology study also suggests that obesity is a risk factor for pancreatic cancer [6]. Based on a recent study, fatty infiltration in the pancreas may increase the risk of pancreatic ductal adenocarcinoma beyond the effect of obesity alone [7]. The problem with the organ location needs the more accurate imaging diagnostic procedures, such as abdominal MRI or even Endoscopic Ultrasound (EUS). The fat content of the pancreas also can be estimated by 3D two point Dixon techniques. However, in population or medical checkup settings, it would be unpractical and also costly to examine the pancreas [8–10].

Since the clinical implication of fatty pancreas is still a mater of debate, especially in countries where alcohol consumption is not an issue, therefore, this study was aimed to evaluate the presence of fatty pancreas and estabslihed its associated risk factors.

## Method

### Study design and subject

The study design was an analytical cross-sectional study among medical check-up patients in Medistra Hospital which has been approved from the Medistra hospital’s local ethics committee between January and December 2013. Inclusion criteria was adult patients aged more than 18 years, no serious illness at the time of examination, having routine laboratory check-up for liver function test, fasting plasma glucose (FPG) levels, and lipid profile, and underwent abdominal ultrasound assessment. None of the patients had a history of significant alcohol drinking (<20 g/day). Patients were excluded from analyses if the pancreas was not visualized on ultrasound or laboratory data was incomplete. Risk factors of NAFPD tested were gender, age group, history of diabetes, body mass index (BMI), systolic and diastolic blood pressures (BP), FPG, triglycerides, total cholesterol, low-density lipoprotein (LDL)-cholesterol, and high-density lipoprotein (HDL)-cholesterol levels. Diabetes mellitus (DM) is diagnosed based on clear history from patient’s interview and the blood glucose database. The metabolic data parameters were collected as the parameters which become our hospital medical check-up standard examination. The minimum sample size for estimating one population proportion at an anticipated proportion of 50 %, 99 % level of confidence and 5 % margin of error was 664 patients.

### Diagnosis of fatty pancreas

Fatty pancreas or pancreas lipomatosis was diagnosed using abdominal ultrasound technique performed by one experienced radiologist in the hospital using a high-resolution ultrasound machine equipped with a 3.5 MHz convex-array probe (LOGIC S7, GE System, US). The radiologist who performed the ultrasound examinations was blinded to the laboratory data; the results were evaluated by another experienced radiologist to ensure unbiased evaluation. Pancreatic echogenicity was compared to the liver echogenicity at the same depth on a longitudinal scan taken near the abdominal midline [11]. If the liver also showed increased echogenicity, comparison was also made with the renal cortex. Diagnosis of pancreas lipomatosis was established if there is increased echogenicity of the pancreas over the liver or renal cortex.

### Laboratory tests

All subjects underwent laboratory tests for standard medical check-up consisting of complete peripheral blood test, liver function test, fasting plasma glucose and lipid profiling. These tests were performed after an overnight fast for a minimum of 10 h.

### Statistical analyses

Characteristics of the study subjects were presented descriptively. Bivariate analyses between the presence of NAFPD and risk factors were performed using the Chi-square test. A *p* value of less than 0.05 was considered significant. Multivariate logistic regression was used to find independent risk factors for NAFPD. Statistical analysis was done using SPSS version 17.0.

## Results

### Characteristics of the study subjects

There were 1054 cases enrolled in the analysis; 720 (68.3 %) of them were men. The mean age was 43.1 ± 12.19 years old. Other characteristics were summarized in Table 1. Pancreas was non-visualized in 153 (14.5 %) cases. In the remaining 901 cases, fatty pancreas was found in 315 (35 %) patients.

**Table 1:** Characteristics of the study subject (*N* = 1054)

Characteristic	Mean (SD)	N	%
Male gender		720	68.3
Age (years)	43.1 ± 12.19		
Age >35 years old		723	68.6
Body mass index (kg/m^2^)	24.9 ± 3.96		
Systolic blood pressure (mmHg)	120 ± 37.2		
Diastolic blood pressure (mmHg)	75 ± 9.9		
Fasting plasma glucose (mg/dL)	96.7 ± 24.69		
Triglyceride levels (mg/dL)	127.1 ± 89.15		
Total cholesterol levels (mg/dL)	205.4 ± 59.0		
LDL-cholesterol	133.2 ± 35.29		
HDL-cholesterol	51.1 ± 12.87		
NAFLD			
No		516	49.0
Yes		538	51.0

### Associations among metabolic risk factors and fatty liver

The presence of fatty pancreas were significantly associated with male gender, age >35 years, higher systolic and diastolic blood pressures, fasting blood glucose >100 mg/dL, triglycerides, total and LDL-cholesterol, and lower HDL cholesterol levels (Table 2).

**Table 2:** Associations among risk factors and the presence of fatty pancreas (*n* = 901)

Risk Factor	Fatty pancreas		Odds ratio	*p* value
	Yes (*n* = 315)	No (*n* = 586)	(95 % CI)	(χ^2^)
Male sex (*n* = 574)	228 (72.4 %)	346 (59.0 %)	1.818 (1.351–2.446)	<0.001
Age > 35 years (*n* = 612)	268 (85.1 %)	344 (58.7 %)	4.011 (2.824–5.697)	<0.001
Systolic BP ≥ 130 mmHg (*n* = 201)	99 (31.4 %)	102 (17.4 %)	2.175 (1.580–2.994)	<0.001
Diastolic BP ≥ 85 mmHg (*n* = 111)	54 (17.1 %)	57 (9.7 %)	1.920 (1.286–2.866)	0.001
Body mass index ≥ 25 kg/m^2^ (*n* = 390)	206 (65.4 %)	184 (31.4 %)	4.129 (3.088–5.520)	<0.001
Type 2 diabetes mellitus (*n* = 62)	31 (9.8 %)	31 (5.3 %)	1.954 (1.164–3.280)	0.010
FPG ≥ 100 mg/dL (*n* = 187)	99 (31.4 %)	88 (15.0 %)	2.594 (1.867–3.603)	<0.001
Triglycerides ≥ 150 mg/dL (*n* = 226)	105 (33.3 %)	121 (20.6 %)	1.921 (1.412–2.615)	<0.001
Total cholesterol ≥ 200 mg/dL (*n* = 491)	203 (64.4 %)	288 (49.1 %)	1.875 (1.415–2.486)	<0.001
LDL-C ≥ 100 mg/dL (*n* = 742)	275 (87.3 %)	467 (79.7 %)	1.752 (1.189–2.582)	0.004
HDL-C < 40 (M) or < 50 (F) mg/dL (*n* = 194)	86 (27.3 %)	108 (18.4 %)	1.662 (1.202–2.298)	0.002

*BMI* body mass index, ***FPG*** **fasting plasma glucose**, *LDL-C* low-density lipoprotein cholesterol*, HDL-C* high-density lipoprotein cholesterol

### Association between fatty pancreas and fatty liver

Fatty liver was present in 538 (51.0 %) of the total study subjects. Among 901 patients with visualized pancreas, fatty pancreas coexisted with fatty liver in 232 (25.7 %) patients; both were absent in 381 (42.3 %) patients. In addition, fatty pancreas with normal liver was found in 83 patients (16.0 %) and fatty liver with normal pancreas was seen in 205 (39.4 %) patients. There was a significant association between fatty pancreas and fatty liver (OR: 5.195; 95 %CI: 3.838-7.032; *p* < 0.001).

**Fig. 1:**
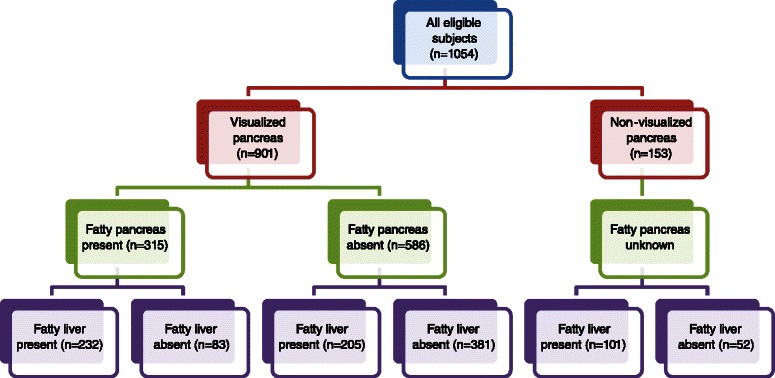
Frequency of fatty pancreas in relation to the presence of fatty liver among patients with visualized pancreas

When the 381 patients with neither fatty liver nor fatty pancreas were excluded, fatty pancreas coexisted with fatty liver in 232 patients (44.6 %). This number can be brokendown further, revealing that fatty pancreas was found in 53.1 % of patients with fatty liver whereas fatty liver can be found in 73.7 % of patients with fatty pancreas (Fig. 1).

### Association between fatty pancreas and diabetes mellitus

Type 2 diabetes mellitus was present in 62 (6.9 %) of the study subects and fatty pancreas was detected in 31 (50 %) of them. Statistically, diabetes has significant associated with fatty pancreas (OR 1.953; 95 % CI: 1.164–3.280; *p* < 0.001). However, when entered into the multivariate analyses; diabetes failed to show a significant association, suggesting that it was a confounding factor rather than an independent risk factor.

## Discussion

### Characteristics of the study subjects

With a detection rate of 35 %, our study showed that the prevalence of nonalcoholic fatty pancreas is high among adult patients underwent routine medical check-up. A recent study involving 8097 subjects underwent health check-up in Taiwan found only 16 % prevalence of fatty pancreas detected by abdominal ultrasound [12]. On the contrary, a Korean study found that 67.9 % of 293 subjects visiting an obesity clinic had fatty pancreas detected by abdominal ultrasound. Larger sample size allows more valid generalization into the general population; therefore, the estimated prevalence of fatty pancreas in the real community may be best represented by the Taiwanese study. The subjects of our study might give an over-estimate result because most of our hospital’s medical check-up subjects came from high social economic groups. Our study may also not represent the true Indonesian population since the patients were recruited from a referral private hospital. However, the high prevalence of NAFPD has given a new insight in patient’s follow up and screening as the risk of malignancy might also be increasing. A more accurate estimation might be shown by a recent study among 685 Hong Kong Chinese healthy volunteers; the prevalence of fatty pancreas detected by fat-water magnetic resonance imaging was 16.1 % [13].

### Association between fatty pancreeas and metabolic risk factors

Our results showed significant association between fatty pancreas and metabolic risk factors (Table 2). Table 3 showed independent risk factors to predict fatty pancreas. These results were consistent with previous epidemiological report in Hong Kong Chinese population [10]. The OR of DM and other variables mostly around the value of 2.0; therefore although systolic BP have higher OR, the difference with DM or triglycerides can be considered not too wide. However, the number of subjects with DM is relatively small compared to other variables. It could affect the statistical calculation of OR and may explain the slight difference with systolic BP. From the statistical point of view, clinical value of an OR occurs when it is more than 1.5. Compared to age or BMI (which have OR >4.0), the ORs of DM and systolic BP are much weaker, it’s also the same with other variables with OR value less than 2.0. From the metabolic point of view, DM is considered as part of metabolic syndrome with could confound other variables within the metabolic markers group (such as cholesterol or TG). On the other hand, blood pressure is a hemodynamic marker. High blood pressure in metabolic syndrome is a consequence of insulin resistance with various underlying mechanisms [14]. Therefore, higher OR of systolic BP might reflect that is more important as risk factor than DM or other lipid parameters.

**Table 3:** Independent risk factors to predict fatty pancreas (*N* = 901)

Risk factor	β	SE (β)	β/SE	OR_adj_	95 % CI	*p* value
Age > 35 years	1.077	0.191	5.639	2.936	2.020–4.267	<0.001
BMI ≥ 25 kg/m^2^	1.261	0.154	8.188	3.529	2.607–4.775	<0.001
FPG ≥ 100 mg/dL	0.531	0.183	2.902	1.701	1.189–2.434	0.004
Total cholesterol ≥ 200 mg/dL	0.491	0.157	3.127	1.634	1.201–2.223	<0.001
Constant	−2.423					

*BMI* body mass index, ***FPG*** **fasting plasma glucose**

### Association between fatty pancreas and fatty liver

Fatty liver was reported as a predictor of “hyperechogenic pancreas” seen during endoscopic ultrasound [15]. A postmortem study collected from 80 cadavers found that total pancreatic fat was a significant predictor of NAFLD, but no correlation was found between pancreatic fat and NAFLD activity score after corrected for body mass index [16]. Our study showed significant correlation between fatty pancreas and fatty liver, however whether with these two entities might doubling the risk of malignancy is still unknown.

Despite the presence of statistical association, the pathophysiology underlying both conditions might be coincidence rather than causative. Both fatty pancreas and fatty liver are significantly associated with metabolic risk factors due to excessive energy intake.

### Association between fatty pancreas and diabetes mellitus

Based on our study, even though there is a significant association between the presence of fatty pancreas and diabetes mellitus, however the association was not observed in the multivariate analysis. Different result was reported among 7464 subjects underwent physical check-up in Taiwan. This might be due to the smaller size effect in the subgroup analysis. The investigators did found an independent association between fatty pancreas and diabetes (OR: 1.379; 95 %CI: 1-047-1.816) [17]. However, this study was not designed to establish a direct causal relationship between diabetes mellitus and fatty pancreas or *vice versa* and should be interpreted as such. The complex factor in insulin resistance pathway might play an important factor. High free fatty acid (FFA) will induce the accumulation of pancreatic fat and the fat deposition will disrupt the beta cell function. The beta cell dysfunction results in unmet relative insulin need to maintain optimal glycemic control and in combination with insulin resistance at the periphery synergistically contributes to the long-term hyperglycemia. This is an important pathway for the development of diabetes mellitus. In return, the insulin resistance condition where induced by FFA source in the pancreas might induce the chronic inflammation stage where this can become a malignancy risk [18]. With these findings, we might also need to screen our diabetes patients for pancreatic malignancy risk.

This study has several limitations. First, the study was designed as a cross-sectional study which cannot prove causal link between fatty pancreas and the associated factors. However, the large sample size in this study could provide generalization to the larger population of interest. Body mass index (BMI) as a risk for pancreatic lipomatosis might be a confounding factor to detect the presence of fatty pancreas. Second, the data about diabetes medication not being reported based on our MCU standard examination record. But this study has given the importance of metabolic factors associated with the presence of NAFPD. Third, the need to differentiate between simple steatosis and steatosis with inflammation in the pancreas has not been elucidated. The transabdominal ultrasound study might not be the best of choice, but ultrasound is still a qualified technique in an experienced hand, and also cheaper when compared to MRI in the community setting. Further study with ethical consideration will be needed to perform pancreas biopsy using EUS study.

## Conclusion

The prevalence of NAFPD in Indonesia is high and it is strongly correlated with other metabolic conditions. The clinical significance of routine fatty pancreas screening needs to be included in our clinical practice. However, it would need further investigation about the long standing condition of fatty pancreas and the usefulness of pancreatic biopsy to see the possibility of disease progression.

## Abbreviations

NAFPD: Non-alcoholic Fatty Pancreas Disease
NAFLD: Non-alcoholic Fatty Liver Disease
EUS: Endoscopic Ultrasound
BMI: Body Mass Index
FPG: Fasting Plasma Glucose
TG: Triglyceride
LDL: Low Density Lipoprotein
HDL: High Density Lipoprotein
BP: Blood Pressure
